# Integrated Dissection of lncRNA-Perturbated Triplets Reveals Novel Prognostic Signatures Across Cancer Types

**DOI:** 10.3390/ijms21176087

**Published:** 2020-08-24

**Authors:** Yunzhen Wei, Limeng Zhou, Yingzhang Huang, Dianjing Guo

**Affiliations:** School of Life Science and State Key Laboratory of Agrobiotechnology, The Chinese University of Hong Kong, Hong Kong 999077, China; weiyunzhen@outlook.com (Y.W.); zhoulm1993@163.com (L.Z.); 1155118996@link.cuhk.edu.hk (Y.H.)

**Keywords:** lncRNA, lncRNA-perturbated triplets, pan-cancer analysis, prognostic signatures

## Abstract

Long noncoding RNA (lncRNA)/microRNA(miRNA)/mRNA triplets contribute to cancer biology. However, identifying significative triplets remains a major challenge for cancer research. The dynamic changes among factors of the triplets have been less understood. Here, by integrating target information and expression datasets, we proposed a novel computational framework to identify the triplets termed as “lncRNA-perturbated triplets”. We applied the framework to five cancer datasets in The Cancer Genome Atlas (TCGA) project and identified 109 triplets. We showed that the paired miRNAs and mRNAs were widely perturbated by lncRNAs in different cancer types. LncRNA perturbators and lncRNA-perturbated mRNAs showed significantly higher evolutionary conservation than other lncRNAs and mRNAs. Importantly, the lncRNA-perturbated triplets exhibited high cancer specificity. The pan-cancer perturbator *OIP5-AS1* had higher expression level than that of the cancer-specific perturbators. These lncRNA perturbators were significantly enriched in known cancer-related pathways. Furthermore, among the 25 lncRNA in the 109 triplets, lncRNA *SNHG7* was identified as a stable potential biomarker in lung adenocarcinoma (LUAD) by combining the TCGA dataset and two independent GEO datasets. Results from cell transfection also indicated that overexpression of lncRNA *SNHG7* and *TUG1* enhanced the expression of the corresponding mRNA *PNMA2* and *CDC7* in LUAD. Our study provides a systematic dissection of lncRNA-perturbated triplets and facilitates our understanding of the molecular roles of lncRNAs in cancers.

## 1. Introduction

Transcriptomic studies have shown that the human genome contains an abundance of non-coding RNAs that are often multiexonic and polyadenylated but lack obvious protein-coding capacity [1,2]. Long noncoding RNAs (lncRNAs, length >200 nt) are a large class of noncoding RNAs involved in various disease-related biological processes, such as affecting the immune system [3], perturbating cell cycle process [4,5] and regulating multiple cancer-related pathways [6,7]. Notably, an increasing number of studies indicate that “lncRNAs regulate microRNA(miRNA)/mRNA axes (which means lncRNAs regulate the expression of mRNA via miRNA)” is one of the most important regulation ways of lncRNAs [8,9]. The regulatory roles of the lncRNA/miRNA/mRNA axes have been extensively explored in different cancer types. For example, *HOTAIR* is known as an oncogenic lncRNA in various cancer types [10] which affects cancer progress via forming *HOTAIR*/miRNA/mRNA axes. It was identified as an lncRNA-directed biomarker in gastric cancer that regulates *HER2* by sponging *miR-331-3p* [11]. Similarly, Ding et al. [12] reported that estrogen receptor β interacts with the *HOTAIR*/miRNA/mRNA axis and promotes the progression of renal cell carcinoma. These evidences provide new insights into the lncRNA functions in cancer biology. 

In recent years, with the development of high throughput RNA-sequencing, a number of human disease datasets with large sample sizes, such as, The Cancer Genome Atlas (TCGA) project [13] et cetera became available. Systematic identification of functional lncRNAs by constructing a lncRNA/miRNA/mRNA network was attempted. For example, Song et al. [14] identified novel functional lncRNAs associated with progression and prognosis of cholangiocarcinoma; Fan et al. [15] developed an lncRNA/miRNA/mRNA network to further understand the lncRNA working mechanism and pathogenesis in colorectal cancer. These studies contributed to the understanding of the roles of lncRNA/miRNA/mRNA axes in cancer but still need to be improved. For instance, it is well established that miRNAs could bind to mRNAs and further decrease the expression level of mRNAs in mammals [16]. However, most studies ignored the correlation between miRNAs and mRNAs when they constructed the lncRNA/miRNA/mRNA networks. For example, an lncRNA/miRNA/mRNA network was established for human estrogen receptor-positive and estrogen receptor-negative breast cancer by integrating miRNA-mRNA interaction pairs, miRNA-lncRNA interaction pairs and lncRNA-mRNA co-expression pairs without considering the negative correlation between miRNAs and mRNAs [17]. Moreover, lncRNAs regulate mRNAs through a variety of ways [18], and yet whether all the identified lncRNAs function through the regulatory axes is still questionable. Previous studies have identified hundreds to thousands of functional lncRNAs from lncRNA/miRNA/mRNA networks. Intriguingly, compared to mRNAs, these lncRNAs showed lower expression levels [19]. This raises a question as to whether these lncRNAs are all effective in regulating mRNA expression. As the first step to understanding the role of lncRNAs in cancer, it is necessary to conduct a systematic identification of effective lncRNA/miRNA/mRNA axes.

In this work, we proposed a new framework to identify effective lncRNA-mediated triplets in five cancer types (lower grade glioma, LGG; kidney clear cell carcinoma, KIRC; kidney papillary cell carcinoma, KIRP; lung adenocarcinoma, LUAD; prostate cancer, PRAD). The lncRNAs in triplets, termed as lncRNA perturbators, exhibited significantly higher evolutionary conservation compared to other lncRNAs, suggesting the functional importance of the identified perturbators. Interestingly, we found lncRNA-perturbated triplets showed high cancer specificity, highlighting the broad heterogeneity underlying various cancer types. We also identified a pan-cancer perturbator, lncRNA *OIP5-AS1*, likely functioning universally in various cancer types. Functional analysis further indicated that these lncRNA perturbators were enriched in different roles in various cancer types. Moreover, lncRNA-perturbated triplets associated with patient overall survival were identified as potential novel prognosis signatures. LncRNA *SNHG7* was identified as a stable potential biomarker for LUAD and two independent datasets were used for validation. Taking LUAD as an example, we also proved the overexpression of the identified lncRNA perturbators enhanced the expression of their corresponding mRNAs.

## 2. Results

### 2.1. LncRNA-Perturbated Triplets Across 5 Cancer Types

**Figure 1:**
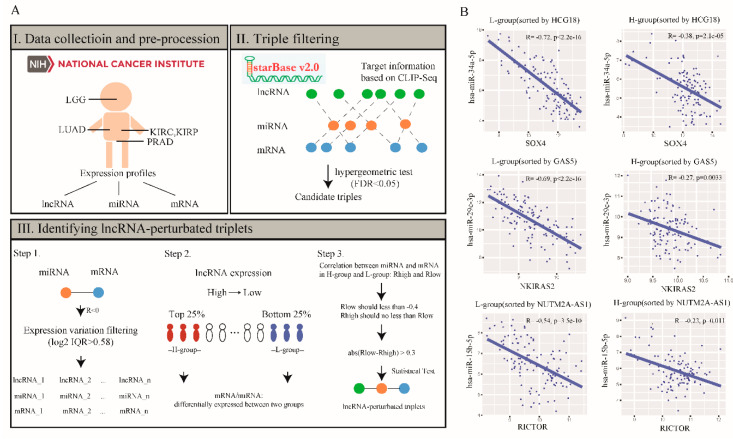
The overview of identification of lncRNA-perturbated triplets in cancer. (**A**) Workflow. The process mainly involved the following three steps: (I) Data collection and pre-procession; (II) collecting Ago CLIP-supported miRNA-target interactions and filtered significant triplets for further analysis by hypergeometric test (FDR < 0.05); (III) identifying lncRNA-perturbated triplets. First, miRNA-mRNA pairs with negative correlation (Spearman′s rank correlation coefficient (SCC): R < 0, *p* < 0.05) and lncRNAs, miRNAs and mRNAs with expression variations across samples (log2 IQR > 0.58; IQR, interquartile range) were used for further analysis. Second, for each lncRNA, we sorted its samples based on the expression. The corresponding miRNAs and mRNAs should show differential expression between the H-group and the L-group (*p* < 0.05, fold-change >1.5). Third, SCC was used to calculate the correlation between miRNAs and mRNAs in the H-group and the L-group, respectively. R value is set as less than –0.4 in the L-group (Rlow) and no less than Rlow in the H-group (Rhigh). In addition, only miRNA-mRNA pairs with the absolute value of difference between Rlow and Rhigh >0.3 were selected as candidate lncRNA-perturbated triplets. (**B**) Some examples of the correlation between the expression of miRNAs (y-axes) and mRNAs (x-axes) in L-group and H-group sorted by *HCG18*, *GAS5* and *NUTM2A-AS1*, respectively, in LGG. Lower grade glioma, LGG; kidney clear cell carcinoma, KIRC; kidney papillary cell carcinoma, KIRP; lung adenocarcinoma, LUAD; prostate cancer, PRAD.

By integrating miRNA-target information and matched expression profiles, we investigated the landscape of lncRNA-perturbated triplets across five cancer types. The process mainly involved the following three steps (Figure 1): (i) Since low tumor purity may influence the results of genomic analysis [20], we first filtered 1,370 high tumor purity patient samples from the five cancer types and collected their matched expression profiles (lncRNA, miRNA and mRNA). (ii) We then collected Ago CLIP-supported miRNA-target interactions and filtered significant triplets for further analysis by hypergeometric test. (iii) LncRNA-perturbated triplets were then identified through the dynamic change among lncRNA, miRNA and mRNA. For each lncRNA, we sorted its samples based on the expression. The Spearman′s rank correlation coefficient (SCC) was used to calculate the correlation between miRNAs and mRNAs in the H-group and the L-group respectively. Figure 1B showed some examples of the correlation between miRNAs and mRNAs in L-group and H-group sorted by *HCG18*, *GAS5* and *NUTM2A-AS1*, respectively, in LGG. Only miRNA-mRNA pairs with the absolute value of difference between Rlow and Rhigh >0.3 were selected as candidate lncRNA-perturbated triplets. Finally, 100 perturbations were performed to test the significance of the identified triplets. As a result, a total of 109 lncRNA-perturbated triplets were identified in the five cancer types (including 12 lncRNA, 25 miRNAs and 94 mRNAs, Table S2). In addition, by integrating the experimentally supported lncRNA-disease association data derived from LncRNADisease v2.0 [21], we found that these lncRNA perturbators were significantly enriched for known disease-related lncRNAs (The overlap between 12 lncRNA perturbators and disease-related lncRNAs with 13,869 lncRNAs as background [22]: *p* = 0.0004, hypergeometric test). For example, perturbator *TUG1* was identified in LUAD and is a known LUAD-related lncRNA. Previous study has reported that *TUG1* expression was induced by *p53*, and *TUG1* knockdown significantly promoted cell proliferation in human non-small cell lung cancer [23]. This observation further suggested the associations of lncRNA perturbators with cancer.

### 2.2. LncRNA Perturbators Show Significantly Higher Evolutionary Conservation Than the Other lncRNAs Bulleted Lists Look Like This:

**Figure 2:**
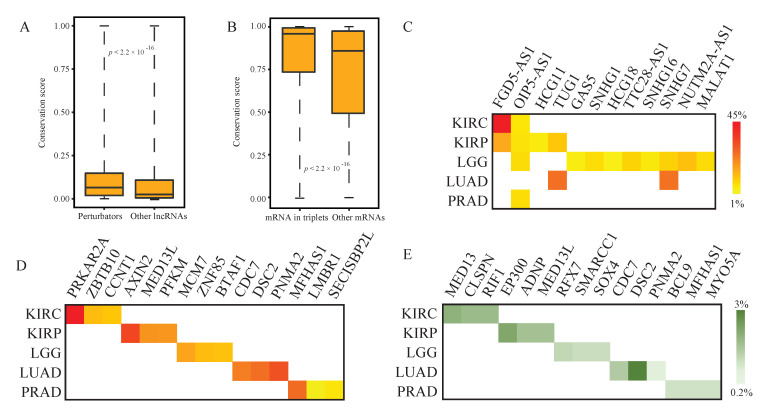
LncRNA perturbators show significantly higher evolutionary conservation than the other lncRNAs. (**A**) The evolutionary conservation score of lncRNA perturbators and other lncRNAs. (**B**) The evolutionary conservation score of lncRNA-perturbated mRNAs and other mRNAs. (**C**) SCNA (somatic copy number alternations) frequency of lncRNA perturbators in 5 cancer types. (**D**) LncRNA-perturbated mRNAs with highest SCNA frequency (top 3) in each cancer type (the same scale bar as (**C**)). (**E**) Non-silent mutation frequency of lncRNA-perturbated mRNAs. Lower grade glioma, LGG; kidney clear cell carcinoma, KIRC; kidney papillary cell carcinoma, KIRP; lung adenocarcinoma, LUAD; prostate cancer, PRAD.

Evolutionary conservation almost always indicates functionality [24]. Here, our results showed that the lncRNA perturbators exhibited significantly higher evolutionary conservation relative to other lncRNAs (Figure 2A). Similarly, lncRNA-perturbated mRNAs also showed a remarkably higher evolutionary conservation than other mRNAs (Figure 2B), highlighting the functional importance of the identified perturbators and perturbated mRNAs. Moreover, we investigated the genomic alterations, including somatic copy number alternations (SCNAs, GISTIC 2.0 [25]) and non-silent somatic mutations [26] of lncRNA perturbators and lncRNA-perturbated mRNAs. We found four paired lncRNA-mRNA exhibiting a substantially high SCNA frequency in cancers (Figure 2C,D), including lncRNA *FGD5-AS1* and *PRKAR2A* (KIRC, 44.2% and 44.4%), lncRNA *TUG1* and *CDC7* (LUAD, 25.9% and 23.6%), lncRNA *TUG1* and *DSC2* (LUAD, 25.9% and 26.1%) and lncRNA *SNHG7* and *PNMA2* (LUAD, 25.4% and 31.1%). In addition, 15 mRNAs were detected with non-silent somatic mutation(Figure 2E), including five mRNAs (*MED13L*, *CDC7*, *DSC2*, *PNMA2* and *MFHAS1*) accompanied with high frequency SCNAs. These results suggested that the triplets may be influenced by genomic alternations.

### 2.3. The Diversity of lncRNA-Perturbated Triplets Highlight the Broad Heterogeneity Underlying Various Cancer Types

**Figure 3:**
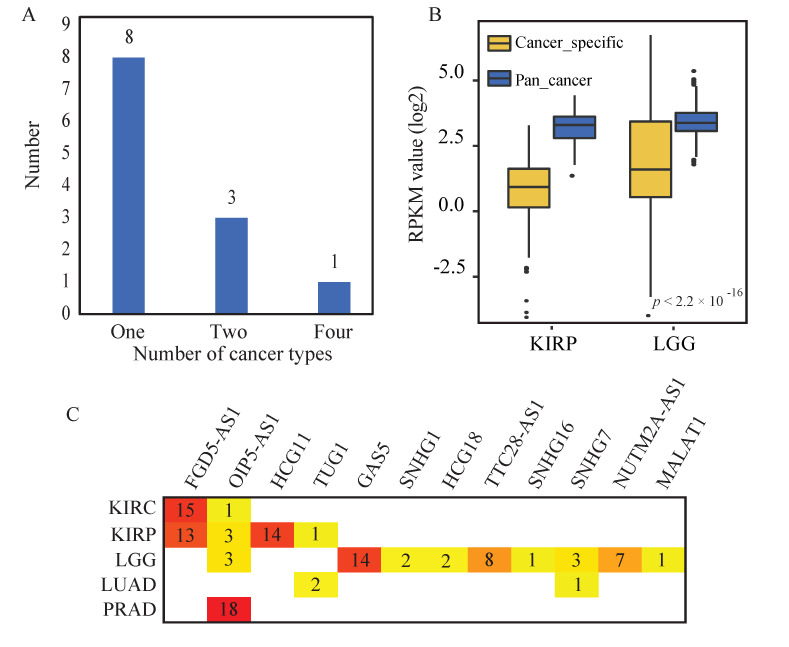
The diversity of lncRNA-perturbated triplets highlight the broad heterogeneity underlying various cancer types. (**A**) The number of lncRNA perturbators that occur in different number of cancer types. (**B**) The expression level of cancer-specific lncRNA perturbators and pan-cancer lncRNA perturbator (pan-cancer lncRNA perturbator, *OIP5-AS1*; cancer-specific lncRNA perturbators, 1 in KIRP and 7 in LGG). (**C**) The number of mRNAs that perturbated by the lncRNA perturbators in 5 cancer types, with deeper color representing larger numbers of mRNAs. Lower grade glioma, LGG; kidney clear cell carcinoma, KIRC; kidney papillary cell carcinoma, KIRP; lung adenocarcinoma, LUAD; prostate cancer, PRAD.

We observed that all of the lncRNA-perturbated triplets were cancer-specific, highlighting the broad heterogeneity underlying various cancer types. Similarly, lncRNA perturbators also exhibited high cancer specificity. Eight out of twelve (66.7%) lncRNAs were detected in only one cancer type (cancer-specific lncRNA perturbators) and the majority of the cancer-specific lncRNA perturbators were observed in LGG (seven out of eight, 87.5%, Figure 3A). In contrast, a lncRNA perturbator (lncRNA *OIP5-AS1*, pan-cancer perturbator) was recurrently detected in four out of the five cancer types. When comparing the differences between the cancer-specific lncRNA perturbators (including one cancer-specific lncRNA perturbator in KIRP and seven cancer-specific lncRNA perturbators in LGG) and the pan-cancer lncRNA perturbator, the pan-cancer lncRNA perturbator showed significantly higher expression than that of the cancer-specific lncRNA perturbators (Wilcoxon test *p*-value < 0.05, Figure 3B). We further summarized the number of mRNAs that were perturbated by the lncRNA perturbators (Figure 3C). LncRNA *FGD5-AS1* regulated a considerable number of mRNAs in KIRC and KIRP, indicating the pivotal role of the perturbator in these two cancer types. 

### 2.4. Enrichment Analysis Elucidates the Critical Functions of the lncRNA Perturbators in Cancer

**Figure 4:**
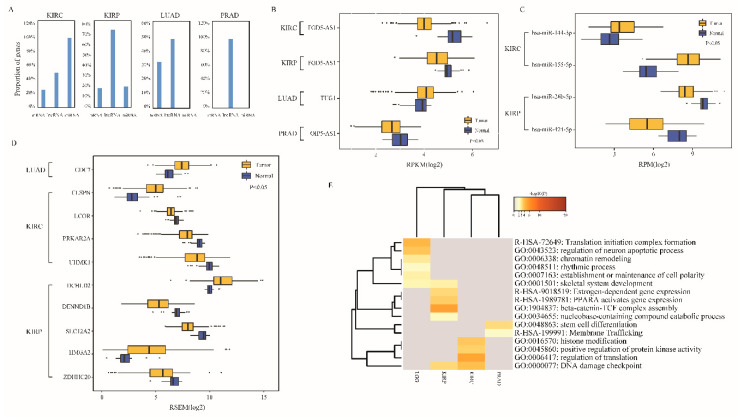
Enrichment analysis elucidates the critical functions of the lncRNA perturbators in cancer. (**A**) Proportion of differentially expressed mRNAs, lncRNAs and miRNAs in 4 cancer types. (**B**–**D**) The differentially expressed lncRNAs, miRNAs and mRNAs in cancer. (**E**) Enrichment result of lncRNA perturbators in 5 cancer types. Each column represents a cancer type and each row represents a Gene Ontology term or a pathway. The color of each table cell corresponds to a different *p*-value (scale bar, −log10(*p*) value). Lower grade glioma, LGG; kidney clear cell carcinoma, KIRC; kidney papillary cell carcinoma, KIRP; lung adenocarcinoma, LUAD; prostate cancer, PRAD.

For the four TCGA cancer types with available normal samples, we found that a large proportion of lncRNA perturbators, miRNAs and lncRNA-perturbated mRNAs exhibited significant differential expression between tumor and normal samples (Figure 4A). Among the three differentially expressed lncRNA perturbators identified in the four cancer types (|log2FC| > 1, FDR < 0.05, Student’s *t*-test), LncRNA *FGD5-AS1* was down-regulated in KIRC and KIRP; lncRNA *TUG1* was up-regulated in LUAD; lncRNA *OIP5-AS1* was down-regulated in PRAD, Figure 4B. Moreover, according to the same criteria, four differentially expressed miRNAs were identified in KIRC and KIRP, with two up-regulated miRNAs and two down-regulated miRNAs (Figure 4C); 10 differentially expressed mRNAs were identified in LUAD, KIRC and KIRP, with four up-regulated mRNAs and six down-regulated mRNAs (Figure 4D). 

To further determine the roles of lncRNA perturbators in cancer, we performed functional enrichment analysis for all the lncRNA perturbators in the five cancer types (using perturbated mRNAs in 109 lncRNA-perturbated triplets). As shown in Figure 4E, these lncRNA perturbators were enriched in key cancer-related pathways and functions. KIRP and KIRC were both enriched in DNA damage checkpoints. Recently, it has been demonstrated that DNA damage can trigger immune response. Tumor DNA damage and repair play important roles in response to immune checkpoint blockade, and clinical researchers are dedicated to advancing reliable DNA damage and repair biomarkers to guide therapy decisions [27]. The result suggested that the lncRNA perturbators in KIRP and KIRC may be involved in immune-related regulation and have potential to serve as predictive biomarkers of immune-directed therapies. Besides, though KIRP and KIRC share similar tissue origins, their lncRNA perturbators were enriched in different pathways and functions (Figure 4E). For example, KIRP was found to be enriched in beta-catenin-TCF complex assembly function. The beta-catenin-TCF complex has a critical role in the Wnt signaling pathway which is over-activated in multiple cancer types [28]; KIRC was found to be enriched in histone modification function, which is a widely known cancer-related function.

### 2.5. LncRNA-Perturbated Triplets with Potential Prognosis Capacity Across Cancer Types

**Figure 5:**
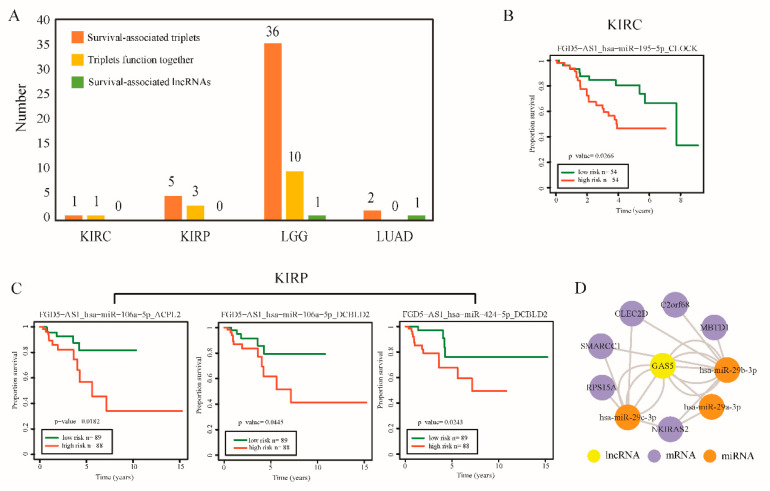
LncRNA-perturbated triplets with potential prognosis capacity across cancer types. (**A**) Number of the identified prognosis-related signatures across cancer types. (Survival-associated triplets: survival-associated triplets identified from 109 lncRNA-perturbated triplets; triplets function together: Triplets associated with overall patient survival though their single factor did not associate with patient survival; survival-associated lncRNAs: survival-associated lncRNAs identified from lncRNA perturbators). (**B**) Kaplan–Meier estimates the overall survival using the triplet “*FGD5-AS1*_*hsa-miR-195-5p*_*CLOCK*” (category: Triplets function together) in KIRC dataset. (**C**) Kaplan–Meier estimates the overall survival using the 3 *FGD5-AS1*-perturbated triplets (category: Triplets function together) in KIRP dataset. (**D**) The *GAS5*-mediated survival-related module (category: Triplets function together) in LGG. Lower grade glioma, LGG; kidney clear cell carcinoma, KIRC; kidney papillary cell carcinoma, KIRP; lung adenocarcinoma, LUAD; prostate cancer, PRAD.

To assess the potential clinical value of lncRNA-perturbated triplets, we used Cox regression analysis to evaluate their independent prognostic significance for overall patient survival. In total, 44 lncRNA-perturbated triplets were identified as novel candidate prognosis signatures across cancer types (Figure 5A and Table S3). Importantly, we found 14 triplets were significantly associated with overall patient survival even though their single factor did not associate with patient survival (“Triplets function together”, Figure 5A). For example, triplet “*FGD5-AS1*_ *hsa-miR-195-5p*_*CLOCK*” could be used to distinguish patient samples with different survival times in KIRC (Figure 5B). However, when univariate risk regression model was applied to single factor of the triplet (*FGD5-AS1*, *hsa-miR-195-5p* or *CLOCK*), no significant association with patient survival was found (*p* = 0.90, 0.67 and 0.08, respectively). Besides, three *FGD5-AS1*-perturbated triplets were also found to be significantly associated with patient survival in KIRP (Figure 5C). Our genomic alteration analysis showed lncRNA *FGD5-AS1* exhibited high frequency SCNA and down-regulated in both KIRC and KIRP. This suggested that lncRNA *FGD5-AS1* tends to have potential function in renal disease and may affect survival of patients with renal disease by forming lncRNA-perturbated triplets. Besides, a *GAS5*-mediated survival-related module was found in LGG (Figure 5D) and was significantly enriched in covalent chromatin modification function (*p* = 0.027). The result revealed that potential interplay may exist among these three factors.

In addition, we identified two novel lncRNA biomarkers in LUAD and LGG, respectively (i.e., *SNHG7* and *NUTM2A-AS1*, *p* = 0.033 and 0.0007, respectively). LncRNA *SNHG7* and *NUTM2A-AS1* have been proven to be associated with cancer process. For example, lncRNA *SNHG7* was indicated in different cancer types such as colorectal cancer [29], pancreatic cancer [30] and lung cancer [31]. However, their prognosis value in LUAD and LGG has never been revealed. Our results contributed to enlarging the potential prognosis signature list for cancer therapy.

### 2.6. Validation of the SNHG7 Signature and lncRNA Perturbators Overexpression in LUAD

**Figure 6:**
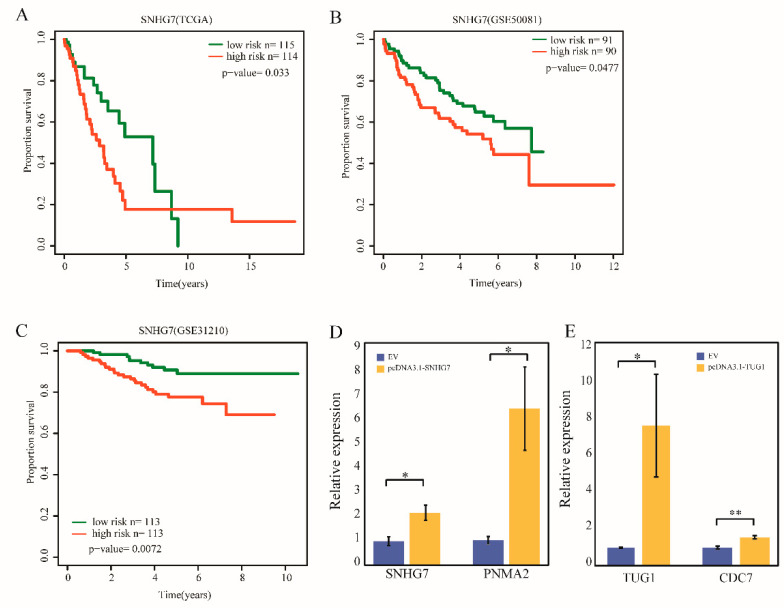
Validate the *SNHG7* signature and overexpress lncRNA perturbators in LUAD. (**A**–**C**) Kaplan–Meier estimates the overall survival using the lncRNA perturbator *SNHG7* in the TCGA LUAD dataset, independent GSE50081 dataset and GSE31210 dataset. (**D**) The expression of *SNHG7* and *PNMA2* was detected in A549 cell line transfected with pcDNA3.1-*SNHG7* and empty vector by qRT-PCR (* *p* < 0.05; ** *p* < 0.01; *** *p* < 0.001; Student *t*-test). (**E**) The expression of *TUG1* and *CDC7* was detected in the A549 cell line transfected with pcDNA3.1-*TUG1* and empty vector by qRT-PCR (* *p* < 0.05; ** *p* < 0.01; *** *p* < 0.001; Student *t*-test. Lung adenocarcinoma, LUAD.

To further validate the prognosis value of the lncRNA perturbators, we took lncRNA *SNHG7*, which was identified from LUAD as an example. In the TCGA dataset, LUAD patient samples could be divided into high- and low-risk groups based on the expression of *SNHG7*, and the two groups showed significantly different survival rate (Figure 6A, *p*-value = 0.033, log-rank test). Next, we collected another two independent datasets to perform the survival analysis. One is the GSE31210 dataset [32], which contains 226 LUAD patient samples from Japan. The other is the GSE50081 dataset [33], which contains 181 LUAD patient samples from Canada. In consistency with the findings derived from the TCGA LUAD dataset, lncRNA *SNHG7* also clearly classified patients into low-risk and high-risk groups with significantly different overall survival rates in both the GSE31210 and GSE50081 datasets (Figure 6B,C, *p*-value = 0.0072 and 0.0477, respectively). This validation further proved that *SNHG7* can be used as a stable prognosis signature for LUAD. For LUAD, we identified three lncRNA-perturbated triplets. To examine the relationship between lncRNA perturbators and lncRNA-perturbated mRNAs, we overexpressed lncRNA *SNHG7* and *TUG1* in the A549 lung adenocarcinoma cell line. The result showed lncRNA *SNHG7* and *TUG1* transfection remarkably enhanced the expression of *PNMA2* and *CDC7*, respectively (Figure 6 D,E). This experimental validation further confirmed our conclusion.

## 3. Discussion

Identification of effective lncRNA-perturbated triplets remains one of the major challenges in cancer biology. Here, we introduced a new framework for the identification of lncRNA-perturbated triplets in multiple cancer types by integrating target information and expression datasets. We found that lncRNA perturbators and lncRNA-perturbated mRNAs showed significantly higher evolutionary conservation than that of the other lncRNAs and mRNAs. It has been reported that frequent genomic alteration in lncRNAs drove the initiation and progression of cancer, for example, increasing telomerase activity [34] and promoting cancer cell proliferation [35]. In our study, we found four paired lncRNA-mRNA exhibiting a substantially high SCNA frequency in cancers, suggesting that genomic alteration may also be involved in such perturbated process. Besides, when applied to five cancer types, the majority of the lncRNA-perturbated triplets identified in this study were cancer-specific. The diversity of the lncRNA-perturbated triplets highlighted the broad heterogeneity underlying various cancer types. On the contrary, we detected a pan-cancer lncRNA perturbator, *OIP5-AS1* with higher expression than that of the cancer-specific lncRNA. The extensive heterogeneity of lncRNA-perturbated triplets also provided the possibility for developing cancer-specific prognosis signatures. In this analysis, 44 lncRNA-perturbated triplets were identified as potential signatures across cancer types. Notably, many lncRNAs, miRNAs and mRNAs may affect patient survival by forming lncRNA-perturbated triplets, highlighting the potential interplay among these factors. Our identification of effective cancer-related lncRNA perturbators and characterization of widespread lncRNA-perturbed triplets across cancer types provide an important foundation for further elucidation of their functional mode in cancer biology. Besides, we identified two novel lncRNA biomarkers in LUAD and LGG (*SNHG7* and *NUTM2A-AS1*). Among them, lncRNA *SNHG7* was validated using two independent datasets (GSE31210 and GSE50081). Finally, to examine the relationship between lncRNA perturbators and lncRNA-perturbated mRNAs, we overexpressed lncRNA *SNHG7* and *TUG1* in the A549 lung adenocarcinoma cell line and experimentally validated their function to remarkably enhance the expression of *PNMA2* and *CDC7* respectively. 

LncRNAs have been established as powerful regulators of gene expression in cancer [36]. Studies suggest that lncRNAs regulate gene expression through diverse mechanisms, such as binding to transcription factors and directing chromatin-modification complexes to specific target regions [37,38]. In recent years, increasing evidences revealed that “lncRNAs regulate miRNA/mRNA axes” was another important function of lncRNAs [39]. Examples include lncRNA *HULC*, one of the most significant regulators in hepatocellular carcinoma whose overexpression inhibits *miR-372* expression and further reduces translational repression of its target gene *CREB* in cell line Hep3B [40]. Some people also called the functioning way that “lncRNAs regulate miRNA/mRNA axes” as competing endogenous RNA (ceRNA) [39]. However, the word “competing” may not be very suitable. Since the majority of lncRNAs is expressed at very low levels compared to mRNAs, the competitive capacity (competing for shared miRNAs) of lncRNAs should be much weaker than that of the mRNAs. In fact, plenty of studies have proven that lncRNAs can indeed regulate the expression of mRNAs via affecting miRNAs/mRNAs axes although the lncRNAs have low abundance [8,9]. This function of lncRNAs is more like “perturbing” than “competing”. Therefore, the lncRNAs identified in our study were named as lncRNA perturbators. Moreover, early studies mainly focused on single lncRNA and lacked efficiency. Although computational studies have been conducted to investigate more lncRNA/miRNA/mRNA axes in different cancer types [41,42], most studies only adopted combined target information and the co-expression between lncRNAs and mRNAs to identify lncRNA/miRNA/mRNA axes. The potential correlation between miRNAs and mRNAs was ignored. Besides, although the negative correlation of miRNA-mRNA pairs was added to the frameworks [43], the required changes among lncRNAs, miRNAs and mRNAs was not considered. Comparing with previous studies, the major advantage of our framework is that we not only utilized the expression data of lncRNAs, miRNAs and mRNAs, the dynamic changes among the three factors were also taken into consideration. Our study dissected the potential lncRNA/miRNA/mRNA axes from a novel perspective: if a lncRNA effectively affects mRNA through miRNA, then the correlation between corresponding mRNA and miRNA should be significantly changed with the change of the lncRNA. In our proposed framework, patient samples were divided into an H-group and an L-group according to the expression of each lncRNA. If the correlation between the miRNA and mRNA change significantly in the H-group and the L-group, the triplets were identified as lncRNA-perturbated triplets.

Certainly, like any other computational approach, our model was also limited by data types and data quality. For example, as the miRNA expression profile of TCGA glioblastoma dataset only contains five patient samples, we therefore could not get enough matched patient samples to do further analysis. Furthermore, only patient samples with high tumor purity were used in our analysis to ensure the accuracy of the results. However, for some cancer types, such as lung squamous cell carcinoma, not enough patient samples were obtained after the filtering process. Further improvement of our framework will rely on additional high-quality patient samples.

## 4. Materials and Methods 

### 4.1. Data Collection and Pre-Processing

The genome-wide lncRNA expression profiles (RPKM profiles) across 5 cancer types were downloaded from TANRIC database [44]. The corresponding mRNA expression profiles (level 3 RNAseq V2 RSEM) and miRNA expression profiles (level 3 RNAseq V2 RPM) were obtained from TCGA project (hub: https://tcga.xenahubs.net). Only samples with matched lncRNA, miRNA and mRNA expression profiles were used in this study. To select samples with high tumor purity, we collected data from a previous report [20,45] to filter our samples (“ESTIMATE” value higher than 0.8). LncRNAs/miRNAs/mRNAs with less than 1 value (log2-transformed) in more than 70% of the samples were removed. The details of the patient samples were summarized in Table S1.

### 4.2. MiRNA-Target Interactions

The miRNA-mRNA and miRNA-lncRNA interaction data were collected from the Starbase V2.0 [46]. The interactions predicted by the 5 prediction programs (including TargetScan [47], miRanda [48], Pictar [49], PITA [50] and RNA22 [51]) were combined for further analysis. MiRNA-lncRNA and miRNA-mRNA pairs sharing the same miRNA were merged into a lncRNA-miRNA-mRNA triplet. Hypergeometric test [52] was used to calculate the significance of shared miRNAs between lncRNAs and mRNAs:

(1) $$P=1-\overset{x-1}{\underset{i=0}{\sum }}\frac{\left(\begin{array}{c} L\\ i \end{array}\right)\left(\begin{array}{c} N-L\\ M-i \end{array}\right)}{\left(\begin{array}{c} N\\ M \end{array}\right)}$$

In this formula, *N* is the total number of miRNAs, and *M* and *L* are the number of miRNAs interacting with lncRNAs and mRNAs, respectively. *X* is the number of miRNAs interacting with both of them. All *p*-values were subject to false discovery rate (FDR) correction and lncRNA-mRNA pairs with FDR < 0.05 were selected for further analysis. 

### 4.3. Identification of lncRNA-Perturbated Triplets

In mammals, miRNAs will destroy the stable structure of mature mRNAs once bound to them, and the expression level of mRNA will be further decreased. Overall, miRNAs and mRNAs should show negative correlation [16]. Here, we used the Spearman correlation coefficient (SCC) to calculate the correlation between mRNAs and miRNAs. The miRNA-mRNA pairs with negative correlation were used for further analysis. A pipeline was designed to identify the lncRNA-perturbated triplets as follows. First, we selected lncRNAs, miRNAs and mRNAs based on their expression variations across samples (log2 IQR > 0.58). Second, for each lncRNA, we sorted its samples based on the expression. The top 25% samples were defined as the H-group, and the bottom 25% were defined as the L-group. The corresponding miRNAs and mRNAs should show differential expression between the H-group and the L-group (miRNA: down-regulated in H-group and up-regulated in L-group; mRNAs: up-regulated in H-group and down-regulated in L-group; *p* < 0.05, fold-change > 1.5). Third, SCC value was used to calculate the correlation between miRNAs and mRNAs in the H-group and the L-group respectively. To ensure that lncRNA effectively perturbs the correlation between miRNA and mRNA, the R value is set as less than −0.4 in the L-group (Rlow) and no less than Rlow in the H-group (Rhigh). In addition, only miRNA-mRNA pairs with the absolute value of difference between Rlow and Rhigh >0.3 were selected as candidate lncRNA-perturbated triplets. Then, referred to previous study [53], the R value of miRNA-mRNA pairs were transformed by Fisher transformation:

(2) $$F\left(R\right)=\frac{1}{2}\ln\frac{1+R}{1-R}$$

The test statistics approximately follows standard normal distribution under the null hypothesis of no difference between the R levels between H-group and L-group. Then a rewiring score is defined (range 0–1) to evaluate the rewiring effect between miRNA and mRNA:

(3) $$\mathrm{rewiring}\;\mathrm{score}=P\left(X\leq \frac{F\left(R_{low}\right)-F\left(R_{high}\right)}{\sqrt{\frac{1}{n_{low}-3}+\frac{1}{n_{high}-3}}}\right),\;X\;\sim \;N\left(0,1\right)$$

Here, a larger rewiring score means strong rewiring effect between miRNA and mRNA; *n_low_* and *n_high_* represent the number of samples in the L-group and the H-group respectively. To confirm the significant difference between L-group and H-group, we randomly sorted patient samples by lncRNA expression and calculated the rewiring score. The process was repeated 100 times. Then we defined a *p*-value for each observed triplet:

(4) $$p\text{-}\mathrm{value}=\frac{n_{random}>s}{m}$$

Here, the *n_random_* > *s* represents the number of times of random rewiring score larger than that of the real condition, and the *m* represents the repeated times. *p*-value < 0.01 was used as the threshold in our study.

### 4.4. Calculation of Evolutionary Conservation Score

The phastCons46way vertebrate evolutionary conservation files for each chromosome were downloaded from the UCSC Genome Browser website [54]. The hg19 annotation information of lncRNAs and mRNAs were obtained from GENCODE [55]. The average phastCons scores for exons were used to evaluate the evolutionary conservation level of lncRNAs and mRNAs.

### 4.5. Functional Analysis

We performed enrichment analysis for the lncRNA-perturbated triplets to investigate their biological roles in cancer. Metascape [56] was used to perform the gene enrichment analysis and adjusted *p*-value < 0.05 was used as the threshold for the significant Gene ontology (GO) terms or pathways.

### 4.6. Evaluation of Prognosis Value of Genes and Triples in Cancers

The corresponding clinical information for 5 cancer types was collected from TCGA. For each lncRNA, miRNA and mRNA, we used a univariate risk regression model to evaluate the association between their expression levels and the patient survival times. The risk factors were identified as the elements (lncRNAs, miRNAs and mRNAs) with plus signs, which means that an increased expression of the elements will lead to decreased survival rates. In contrast, the protective factors were identified as the elements with minus signs, which means that an increased expression of the elements will contribute to increased survival rates. Next, we took into account all three elements and calculated a Risk score (i) for each patient sample [57]:

Risk score = α × exp(lncRNA)_i_ + β × exp(miRNA) _i_ + γ × exp(mRNA)_i_ (5)

where α, β and γ were regression coefficients of lncRNA, miRNA and mRNA, respectively. The mean value of the Risk score was used to divide patient samples into a high-risk group or a low-risk group.

### 4.7. Cell Culture

In this study, cell line A549 was purchased from American Type Culture Collection (ATCC) and cultured in RMPI-1640 (Gibco, Manassas, VA, USA) supplemented with 10% FBS (Gibco) at 37 °C, 5% CO_2_ conditions. 

### 4.8. Transfection

lncRNA was amplified from A549 cell cDNA and cloned into pcDNA3.1. Empty vector and pcDNA3.1-lncRNA was transfected into cells with Lipofectamine 2000 reagent (Invitrogen, Carlsbad, CA, USA), respectively.

### 4.9. RNA Isolation and qRT-PCR

Total RNA was extracted using MiPure Cell/Tissue miRNA Kit (Vazyme, Nanjing, China, 21000) and reverse transcribed (Invitrogen, Carlsbad, CA, USA) at 48 h after cell transfection. Quantitative real-time PCR (qRT-PCR) was performed and GAPDH was used to normalize the expression levels of lncRNA and. The primers were listed as Table S4.

## Supplementary Materials

Supplementary materials can be found at https://www.mdpi.com/1422-0067/21/17/6087/s1. Table S1: Number of tumor samples and normal samples in 5 cancer types, Table S2: The identified 109 triplets in 5 cancer types, Table S3: Survival-related triplets in 5 cancer types, Table S4: Primers for Quantitative real-time PCR (qRT-PCR).
