# Supplementary material for: Integrated Dissection of lncRNA-Perturbated Triplets Reveals Novel Prognostic Signatures Across Cancer Types
Source: Int J Mol Sci. 2020 Aug 24;21(17):6087. doi: 10.3390/ijms21176087 (PMC7503457; doi:10.3390/ijms21176087)
Supplement: Supplementary file 1 [file ijms-21-06087-s001.zip › ijms-853162 suppl/Table S1.docx]

**Table S1**

| **Cancer** | **Tumor samples** | **Normal samples**  **(mRNA)** | **Normal samples**  **(lncRNA)** | **Normal samples**  **(miRNA)** |
| --- | --- | --- | --- | --- |
| KIRC | 108 | 72 | 67 | 70 |
| KIRP | 179 | 33 | 30 | 35 |
| LGG | 476 | NA | NA | NA |
| LUAD | 239 | 59 | 58 | 45 |
| PRAD | 368 | 52 | 52 | 52 |
